# Combined spectroscopy methods and molecular simulations for the binding properties of trametinib to human serum albumin†

**DOI:** 10.1039/c7ra12890h

**Published:** 2018-01-26

**Authors:** Zili Suo, Qiaomei Sun, Hongqin Yang, Peixiao Tang, Ruixue Gan, Xinnuo Xiong, Hui Li

**Affiliations:** College of Chemical Engineering, Sichuan University Chengdu Sichuan China tangpeixiao@126.com lihuilab@sina.com +86 028 85401207 +86 028 85405149

## Abstract

Trametinib is a novel anticancer drug for treating metastatic cutaneous melanoma. The present study probed into the binding of trametinib to human serum albumin (HSA) through spectroscopy methods and molecular simulations. Trametinib could quench the fluorescence of HSA through static quenching which could be probed *via* fluorescence spectroscopy and time-resolved fluorescence. Thermodynamic parameters and docking results indicated that hydrogen bonds and van der Waals forces play crucial roles in this binding process, which exerts almost no effect on the HSA conformation under synchronous fluorescence, three-dimensional fluorescence, circular dichroism spectra, and molecular dynamics simulations. Site marker displacement experiments and molecular docking reveal that trametinib primarily binds to Sudlow site I of HSA. In addition, the trametinib–HSA interaction was hardly influenced by varying amino acid (glutamine, alanine, glycine, and valine) concentrations. This study can provide useful information for the pharmacokinetic properties of trametinib.

## Abstract 2

Probing the binding properties of trametinib to human serum albumin.

## Graphical abstract

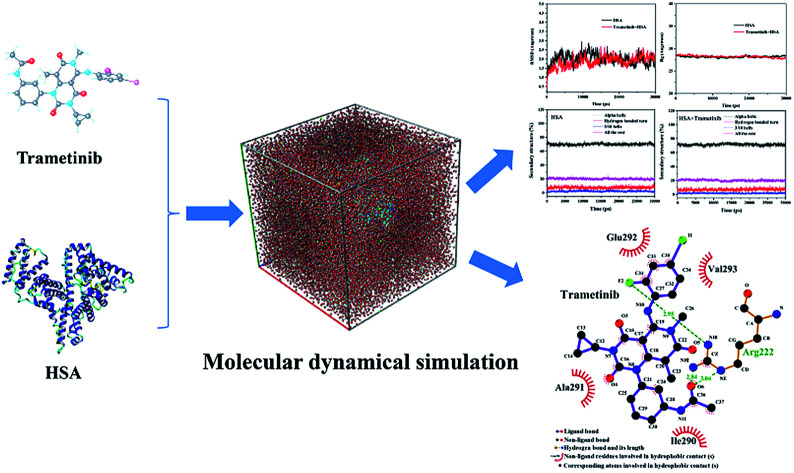

## 1. Introduction

Trametinib (GSK 1120212) is a novel anticancer drug for treating metastatic cutaneous melanoma.^1–3^ It was the earliest listed mitogen-activated, extracellular signal-regulated kinase (MEK) inhibitor worldwide and was first approved by the United States Food and Drug Administration in May 2013.^4–6^ Trametinib was also developed for different cancer types, such as biliary tract cancer, rectal cancer, and metastatic breast cancer.^7^

Trametinib is present mainly in the form of binding with plasma proteins in the blood because of its high binding rate to plasma protein (97.4% (ref. 8)). The largest portion of plasma protein is constituted by human serum albumin (HSA).^9,10^ This protein plays an important role in the transport, absorption, distribution, and metabolism of drugs in the body.^11,12^ Crystallographic analyses have revealed that HSA is a 585-residue protein composed of three domains (I–III). Two subdomains (A and B) comprise an intact domain.^13,14^ Drug binding mainly occurs in the hydrophobic cavity of subdomains IIA and IIIA, namely Sudlow sites I and II correspondingly in HSA.^15,16^ Binding may affect the pharmacokinetic properties of these drugs. Investigating the characteristics of drugs binding to HSA can contribute to an understanding of the pharmacokinetic properties of drugs in the body.^17–19^ The interaction between trametinib and HSA has not been reported to date. Thus, probing the properties of the trametinib–HSA interaction is essential.

In the present work, the trametinib–HSA interaction was explored through several spectroscopy methods, molecular docking, and molecular dynamics simulations. In order to determine the quenching mechanism, fluorescence and time-resolved fluorescence spectroscopy were conducted. The binding parameters were also determined. HSA conformation is important for its function. Thus, synchronous fluorescence, three-dimensional fluorescence, and circular dichroism spectroscopy were performed in this study. To determine the main binding site of HSA for trametinib, site marker displacement experiments, along with docking software were used. Molecular dynamics simulations were applied to investigate the state of the trametinib–HSA complex in solution and possible structural changes of HSA induced by trametinib insertion. Meanwhile, the concentrations of circulating amino acids are different and varied in cancer patients, which may affect trametinib binding to HSA. Therefore, the effects of amino acids on the trametinib–HSA interaction were also studied.

## 2. Experimental

### 2.1 Materials

Fatty acid-free HSA (A1887, ≤0.007% fatty acids) was purchased from Sigma Chemical Company (St Louis, USA). Trametinib (99% purity) was obtained from Shanghai Rongtai Pharmatech Co. Ltd (Shanghai, China). Dansylsarcosine (98%) was acquired from Heowns Biochem Technologies LLC (Tianjin, China). Warfarin sodium (98%), phenylbutazone (99%), ibuprofen (98%), glutamine (99%), alanine (99%), glycine (99%), and valine (99%) were purchased from J&K Scientific Ltd (Beijing, China). Phosphate buffer saline (pH = 7.4) was used in this study.

### 2.2 Instruments and operations

#### 2.2.1 Fluorescence spectroscopy

A Cary Eclipse fluorophotometer (Varian, California, USA) was used to record the fluorescence spectra with excitation and emission slits of 5 and 10 nm, respectively. The excitation wavelength (*λ*_ex_) was set to 280 nm in this study, unless particularly indicated. Synchronous and three-dimensional (3D) fluorescence spectra were also obtained utilizing the same instrument.

In the site marker displacement experiments, the fluorescence spectra of the warfarin–HSA complex were recorded from 340 nm to 500 nm (*λ*_ex_ = 320 nm), and those of the dansylsarcosine–HSA complex from 400 nm to 650 nm (*λ*_ex_ = 350 nm). The final concentrations of HSA and the probe (warfarin/dansylsarcosine) were maintained at 4.0 μM. The concentrations of trametinib, phenylbutazone, and ibuprofen ranged from 0 μM to 20.0 μM in increments of 4.0 μM.

Subsequently, the possible effects of several blood plasma amino acids, *i.e.*, glutamine, alanine, glycine, and valine, on the binding of trametinib to HSA were examined by fluorescence measurements with various concentrations (0–400 μM) of amino acid. The concentrations of HSA and trametinib were maintained at 2.0 μM. Each solution was kept at 298 K for 12 h before the fluorescence measurements.

#### 2.2.2 Correction of inner filter effect

All fluorescence intensities were corrected on the basis of the following equation given the inner filter effect:^20^1
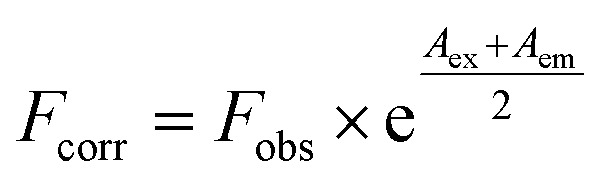
where *F*_corr_ are the corrected fluorescence intensities that are achieved by processing the observed fluorescence intensities (*F*_obs_). A TU-1901 UV-Vis spectrophotometer (Persee, Beijing, China) was used to measure the absorption of the system at the excitation (*A*_ex_) and emission (*A*_em_) wavelengths.

#### 2.2.3 Fluorescence lifetime

A JobinYvon Fluorolog-3 spectrofluorometer (Horiba, LesUlis, FRA) was used to measure the fluorescence lifetime at *λ*_ex_ = 280 nm and *λ*_em_ = 337 nm. The concentration of HSA was 2.0 μM, and the concentrations of trametinib were 0, 4.0, and 8.0 μM.

#### 2.2.4 Circular dichroism (CD) spectra

A Chirascan-plus circular dichroism spectrometer (Applied Photophysics, Surrey, UK) was used to record the CD spectra. The concentrations of trametinib were 0, 4.0, and 8.0 μM, with HSA maintained at 2.0 μM. The spectra were scanned in the range of 200 to 250 nm in triplicate and averaged for graphing and further analyses.

### 2.3 Molecular simulation

#### 2.3.1 Molecular docking

The FlexX docking program interfaced within LeadIT was used to probe the possible conformations of the trametinib–HSA complex.^21,22^ The available crystal structure of HSA (ID: 1H9Z) was acquired from the RCSB Protein Data Bank, and the 3D structure of trametinib was obtained from PubChem (PubChem CID: 11707110). The ligand and receptor were optimized by FlexX to ensure the proper protonation states before docking. The docking area was set as the whole protein. The LigPlot program was used to analyze the simulated results.

#### 2.3.2 Molecular dynamics (MD) simulation

MD simulations were run in YASARA v17.4.17. The force field was assigned as AMBER14.^23^ The AM1-BCC model was used to operate the partial atomic charges of the ligand. The complex was placed in a water box with a size of 100.04 Å × 100.04 Å × 100.04 Å along the *x*, *y*, and *z* axes, and then a periodic boundary condition was applied. The pH was set to 7.4, and the temperature was maintained at 298 K. The system was neutralized by inserting Na^+^ or Cl^−^ counterions. Particle mesh Ewald algorithm was applied to calculate the electrostatic forces, and a cut-off of 8.0 Å was used for the van der Waals forces. The simulation was run with a multiple time step of 1.25 fs and 2.5 fs for intramolecular and intermolecular forces, correspondingly. Data were collected per 10 ps.

## 3. Results and discussion

### 3.1 Fluorescence quenching mechanism

**Fig. 1:**
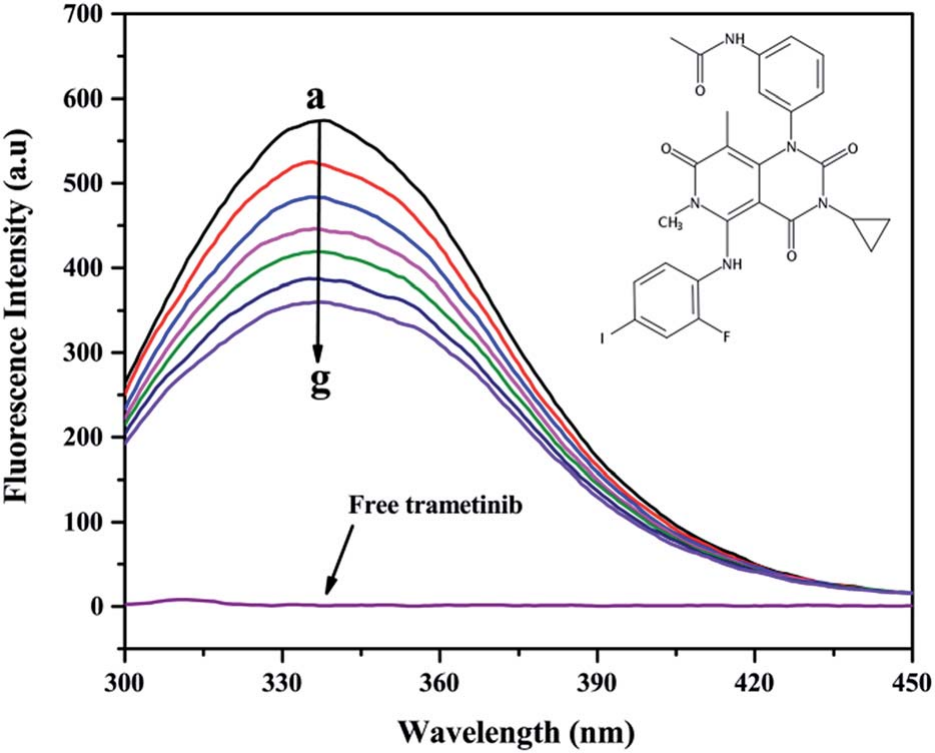
Fluorescence emission spectra of HSA (2.0 μM) with a gradient concentration of trametinib (0–9.0 μM); the fluorescence spectrum of free trametinib was recorded at 9.0 μM. The structure of trametinib is shown at the upper right corner.

The addition of trametinib reduced the fluorescence intensity (Fig. 1) of HSA. The intrinsic fluorescence of HSA is mostly contributed by tryptophan (Trp) and tyrosine (Tyr), which can be affected by the binding of a ligand to HSA.^24^ This result indicated that trametinib binds to HSA.

Several mechanisms (*i.e.*, dynamic quenching and static quenching) explain the fluorescence quenching phenomenon.^25,26^ The Stern–Volmer equation was used to distinguish the fluorescence quenching mechanism in this study:^24^2
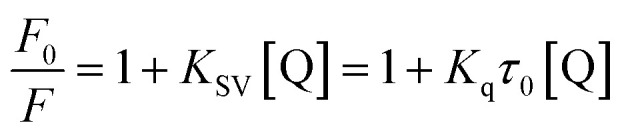
where *K*_SV_ is the Stern–Volmer quenching constant, *F*_0_ is the fluorescence intensity of free HSA, *F* is the fluorescence intensity of HSA after trametinib addition, [Q] is the concentration of trametinib, *K*_q_ is the biomolecular quenching constant, and *τ*_0_ is the lifetime of HSA in the absence of the quencher (*τ*_0_ = 5.073 × 10^−9^ s). The fitting plots are shown in Fig. S1.† The *K*_SV_ (Table 1) values decreased gradually with an increase in temperature. For static quenching, the quenching constant decreased with an increase in temperature.^27^ The maximum scatter collision quenching constant of quenchers with biopolymer is 2.0 × 10^10^ L mol^−1^ s^−1^. *K*_q_ (Table 1) values were greater than 2.0 × 10^10^ L mol^−1^ s^−1^ that indicated the quenching to be static.^28^ The results indicated that the fluorescence quenching mechanism of trametinib with HSA involves static quenching.

**Table 1:** Stern–Volmer quenching constants for the trametinib–HSA system

*T* (K)	*K*_SV_ × 10^4^ (M^−1^)	*R* a	*K*_q_ × 10^13^ (M^−1^ s^−1^)
298	6.702 ± 0.030	0.9979	1.321 ± 0.006
304	5.981 ± 0.017	0.9936	1.179 ± 0.003
310	5.664 ± 0.023	0.9957	1.116 ± 0.004

a The correlation coefficient for the *K*_SV_ values.

Time-resolved fluorescence spectra were used to further verify the quenching mechanism of trametinib with HSA. The tail fitting method was used to process the initial data and the following equation was used to obtain the weighted average fluorescence lifetimes (〈*τ*〉):3
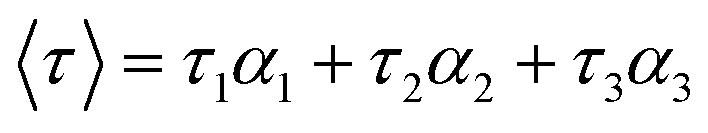
where *α*_1_, *α*_2_, and *α*_3_ are the relative amplitudes; *τ*_1_, *τ*_2_, and *τ*_3_ are the decay times.

**Fig. 2:**
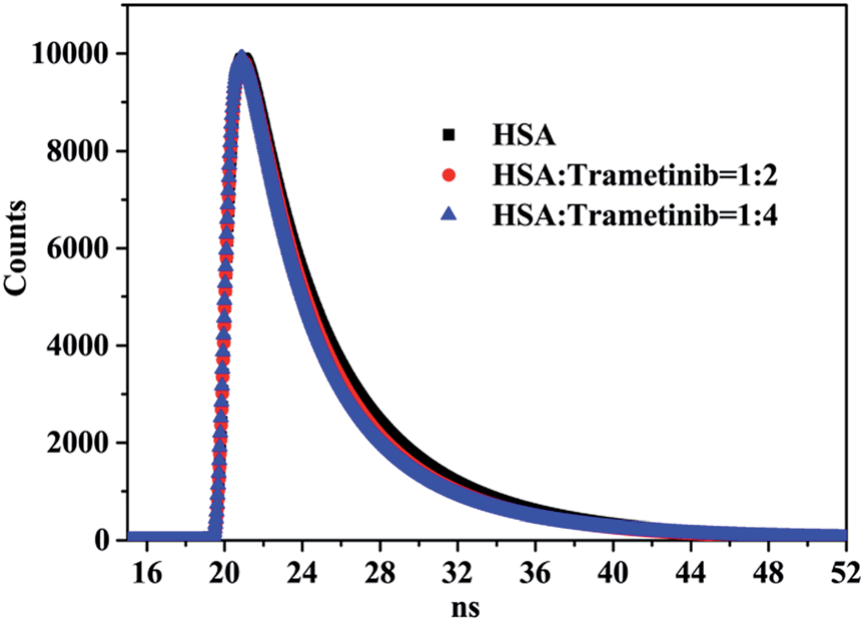
Fluorescence lifetime curves of HSA with different proportions of trametinib.

Fluorescence lifetime data (Table 2 and Fig. 2) indicated that the weighted average lifetime of HSA changed little with the increase in the concentrations of trametinib considering the experimental error. Thus, trametinib quenched the fluorescence of HSA *via* static quenching.^20^

**Table 2:** Fluorescence lifetimes of HSA under different concentrations of trametinib

System	*C*_trametinib_ (μM)	*τ*_1_ (ns)	*τ*_2_ (ns)	*τ*_3_ (ns)	*α*_1_	*α*_2_	*α*_3_	〈*τ*〉 (ns)	*χ* ^2^
	0.0	3.102	0.604	6.796	0.362	0.062	0.576	5.073	1.093
Trametinib–has	4.0	2.926	0.440	6.662	0.403	0.050	0.547	4.846	1.054
Trametinib–HAS	8.0	2.962	0.544	6.648	0.426	0.059	0.515	4.717	1.106

### 3.2 Binding parameters

For static quenching, the number of binding sites (*n*) and binding constant (*K*) were calculated on the basis of the modified Stern–Volmer equation:^29^4
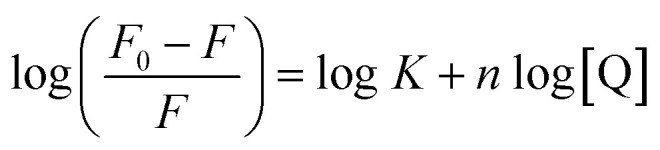


The *K* values (Table 3) indicated that a medium-strength binding occurred between trametinib and HSA. The binding site values at different temperatures were approximately 1 (Table 3), thereby suggesting that HSA contains a high-affinity binding site for trametinib.

**Table 3:** Binding constants *K*, binding sites *n*, and thermodynamic parameters for trametinib–HSA system

*T* (K)	*K* × 10^4^ (M^−1^)	*n*	*R* a	Δ*G* (kJ mol^−1^)	Δ*H* (kJ mol^−1^)	Δ*S* (J mol^−1^ K^−1^)
298	6.826 ± 0.037	1.049	0.9968	−27.53		
304	3.618 ± 0.021	1.024	0.9921	−26.69	−68.93	−138.95
310	2.334 ± 0.031	1.016	0.9937	−25.86		

a The correlation coefficient for the *K* values.

The interactive forces of pharmaceutical molecules with biological macromolecules include hydrogen bond, van der Waals, electrostatic, and hydrophobic interactions. The van't Hoff equation was adopted to determine the interaction forces of trametinib with HSA. Accordingly, the thermodynamic parameters were calculated with the temperature gradient as follows:^30^5
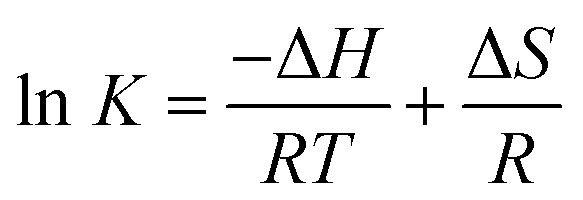
where *R* is the gas constant. The enthalpy change (Δ*H*) and entropy change (Δ*S*) were obtained by fitting the associative binding constants (*K*) and the corresponding temperature (*T*).

The free energy change (Δ*G*) was calculated using the following equation:^30^

6 Δ*G* = Δ*H* − *T*Δ*S* = −*RT* ln *K*

Ross and Subramanian provided a method for determining the force types according to Δ*H* and Δ*S* values.^31^ The Δ*H*, Δ*S*, and Δ*G* values were all negative (Table 3), thereby indicating that trametinib can spontaneously bind with HSA mainly through hydrogen bonding and van der Waals forces.

### 3.3 Effect of trametinib on HSA conformation

**Fig. 3:**
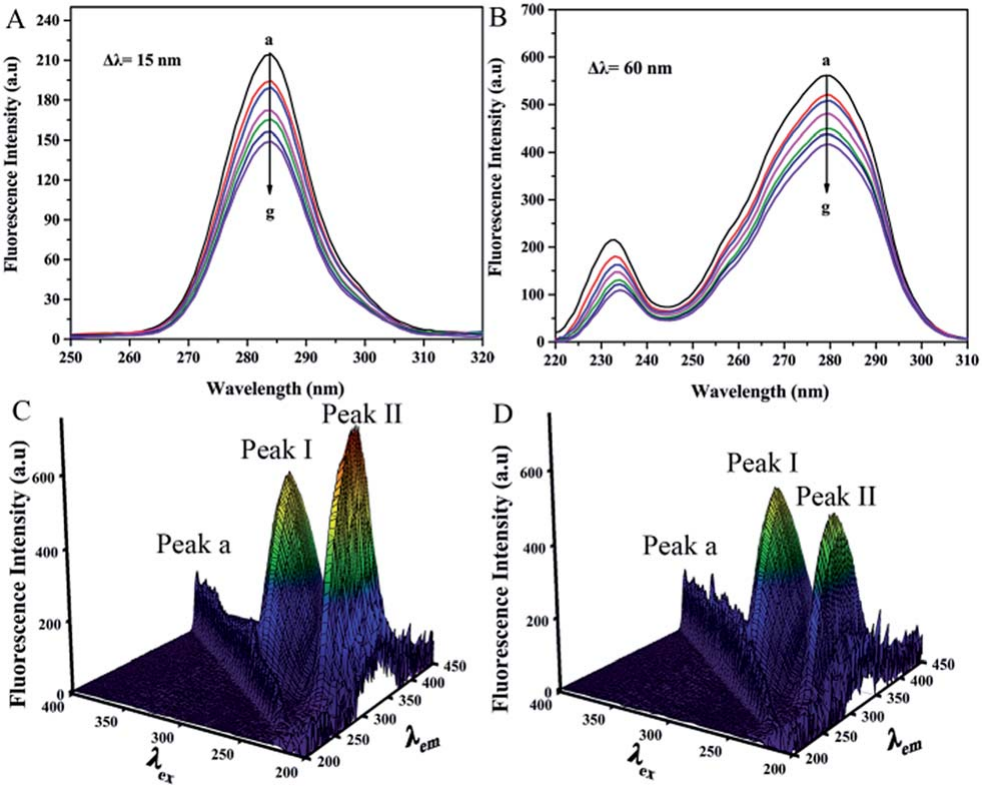
(A and B) Synchronous fluorescence spectra of HSA (2.0 μM) with a gradient concentration of trametinib (0–9.0 μM) at Δ*λ* = 15 nm and Δ*λ* = 60 nm. (C and D) 3D fluorescence spectra of free HSA (2.0 μM) and after addition of trametinib (2.0 μM) with 5 nm increments in excitation wavelength.

Synchronous fluorescence spectroscopy was performed to probe the microenvironmental changes of the chromophore molecules. The method showed favorable selectivity and was beneficial in analyzing changes in the microenvironment of tyrosine (Tyr) (Δ*λ* = 15 nm) and tryptophan (Trp) residues (Δ*λ* = 60 nm).^32^ The position of the maximum emission wavelengths (Δ*λ* = 15 nm and 60 nm) showed no shift within the range of the trametinib concentrations tested (Fig. 3A and B). Therefore, trametinib insertion did not affect the microenvironment polarities of the Tyr and Trp residues in HSA.

3D fluorescence spectroscopy is a common method for studying drug–HSA interactions.^12^ Three peaks were noted using 3D fluorescence spectroscopy (Fig. 3C and D) and marked as Peaks a, I, and II. Peaks I and II are the characteristic signals of aromatic residues in HSA whereas Peak a is the Rayleigh scattering peak. The intensities of Peaks I and II were diminished by trametinib addition because of the fluorescence quenching as a result of trametinib binding to HSA. The positions of Peaks I and II in the two spectrograms were similar (Table 4). Therefore, the local environment of the aromatic residues in HSA was almost unaffected by trametinib insertion.

**Table 4:** 3D fluorescence data of HSA in the absence and presence of trametinib

System	Peak	Peak position [*λ*_ex_/*λ*_em_ (nm/nm)]	Intensity
HSA	I	280/338	587.254
	II	225/340	728.174
HSA–trametinib	I	280/337	514.769
	II	225/338	411.384

**Fig. 4:**
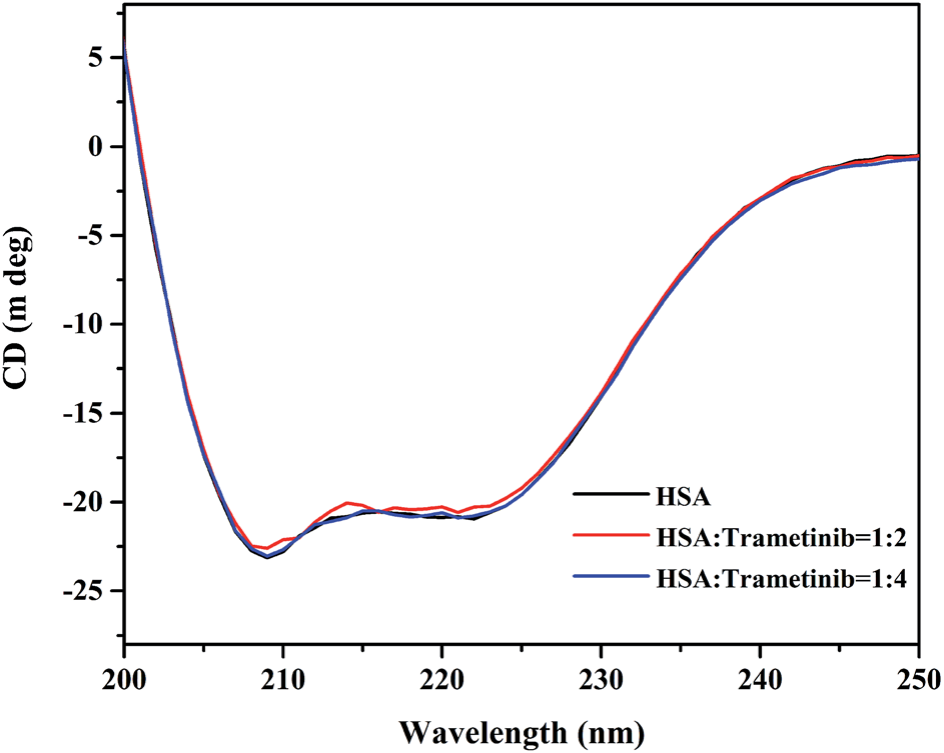
CD spectra of HSA with different proportions of trametinib.

CD spectroscopy is an effective method for monitoring the secondary structural changes in proteins.^25^ In this study, CD spectroscopic analysis was performed to probe the effect of the secondary structural changes in HSA after trametinib addition. The CD spectra (Fig. 4) exhibited two negative ellipticities at 208 and 222 nm, thereby indicating that the secondary structure of HSA is predominantly α-helix. The content of α-helical HSA under different concentrations of trametinib was calculated according to the following equations:7
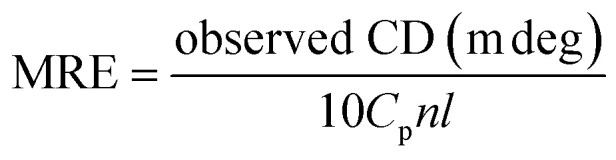
8
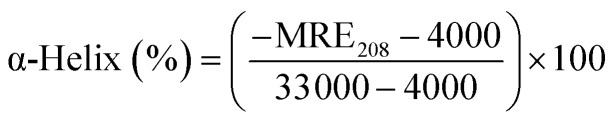
where *C*_p_ is the molar concentration of the protein, *n* is the number of amino acid residues (585 for HSA), *l* is the pathlength of the cell (0.1 cm), and MRE_208_ is the mean residue ellipticity (MRE) value at 208 nm. The calculation results are listed in Table S1† and are all about 53%. The difference in the α-helix content reported here and that of X-ray structural analysis is due to the instrument and method differences.^20^ The shapes and intensity of the CD spectra were almost unchanged with the gradient concentration of trametinib, hence suggesting that the secondary structure of HSA is unaffected by trametinib. This conclusion was further verified by MD simulations as discussed in the following sections.

### 3.4 Determination of the main binding site

**Fig. 5:**
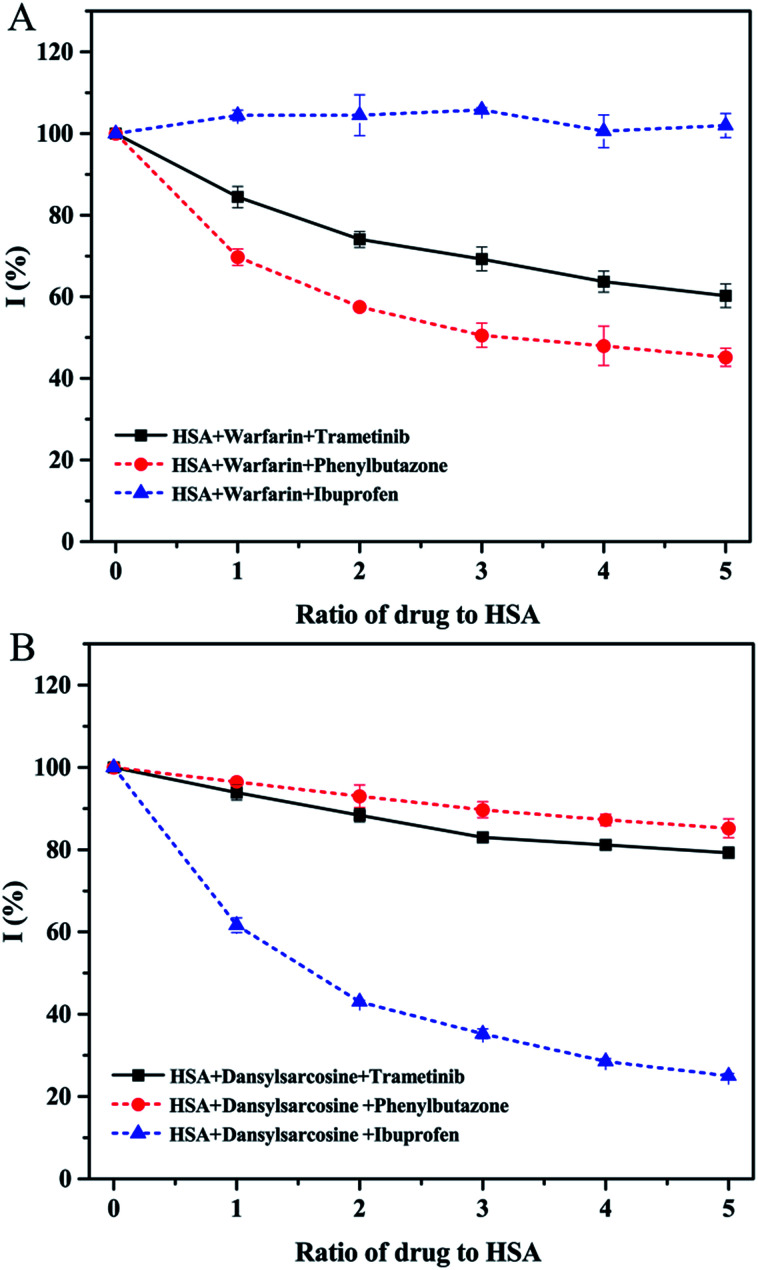
Effect of trametinib (full line) and controls (dotted line) on the fluorescence intensities of warfarin–HSA (A) and dansylsarcosine–HSA (B).

The main binding site in HSA for trametinib was determined by site marker displacement experiments. Several fluorescent probes for HSA were mentioned by Sudlow *et al.* such as warfarin and dansylsarcosine.^33^ Crystallographic analyses have revealed that warfarin binds to Sudlow sites I of HSA and dansylsarcosine binds to Sudlow sites II.^34,35^ Warfarin and dansylsarcosine were used as probes for the two primary sites (Sudlow sites I and II) of HSA in the present study, and changes in the fluorescence of the probes provide measures for drug binding sites.^36,37^ Phenylbutazone and ibuprofen were used as controls, which have been verified to bind with HSA at Sudlow sites I and II, respectively.^38^ The percentage (*I*) of the initial fluorescence is determined using the following equation:9
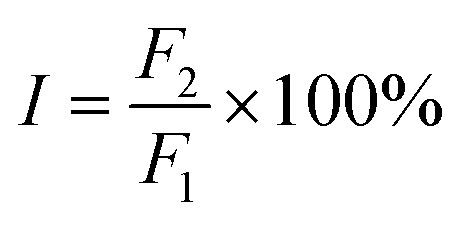
where *F*_1_ is the fluorescence intensity of free probe–HSA, and *F*_2_ is the fluorescence intensity of probe–HSA with the gradient concentration of trametinib or controls. Phenylbutazone and trametinib induced a distinct decline in fluorescence intensity of warfarin–HSA but minimally affected the fluorescence intensity of dansylsarcosine–HSA (Fig. 5). This result indicated that trametinib mainly bound to Sudlow site I, which is the binding site of warfarin and phenylbutazone.

**Fig. 6:**
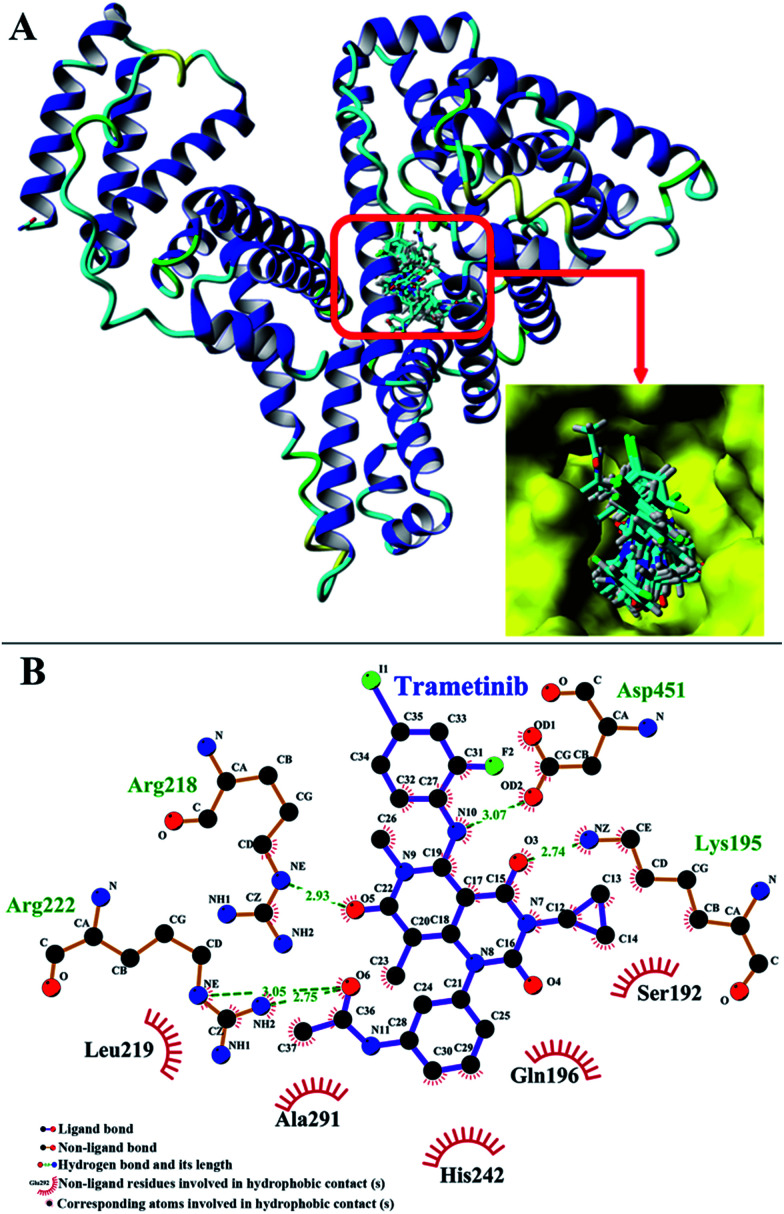
(A) Cluster analysis for the first 20 conformations of trametinib binding to HSA. (B) First binding conformation of trametinib to HSA as indicated by FlexX scores.

Molecular docking was adopted to predict the drug binding sites in the protein. The FlexX docking software was used to probe the binding site of trametinib in HSA. The docking results were ranked by FlexX scores. The first 20 docking results (Fig. 6A) showed that trametinib binds to Sudlow site I, thereby suggesting that Sudlow site I is the preferred binding site for trametinib. These results agree with those of the site marker displacement experiments.

### 3.5 Molecular simulations

The binding model of the trametinib–HSA complex was explored in this study by using the FlexX docking software. The docking results were ranked by FlexX scores and the first ranking model is presented in Fig. 6B. The trametinib molecule connected with four amino acid residues (Lys 195, Arg 218, Arg 222, and Asp 451) by hydrogen bonds and five amino acid residues (Gln 196, Ser 192, Leu 219, His 242, and Ala 291) by hydrophobic interactions. The total hydrogen bond energy was 89.65 kJ mol^−1^ and the total hydrophobic interaction energy was 24.35 kJ mol^−1^. These values indicated that hydrogen bonds played crucial roles, and hydrophobic interactions were also involved in the binding process.

MD simulations were performed to investigate the state of the trametinib–HSA complex in solution, and the possible structural changes of HSA caused by trametinib insertion.^39,40^ YASARA was used to calculate the values of root mean square deviations (RMSD), radius of gyration (Rg), and percentage of secondary structure.

**Fig. 7:**
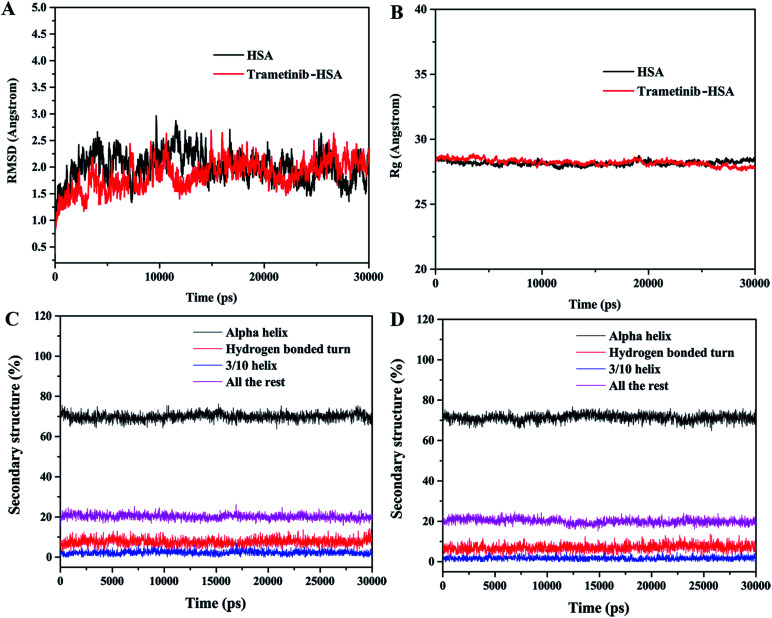
RMSD (A) and Rg (B) of HSA and the trametinib–HSA complex. Secondary structure percentages of HSA in the absence (C) and presence (D) of trametinib in 30 ns MD simulations.

**Fig. 8:**
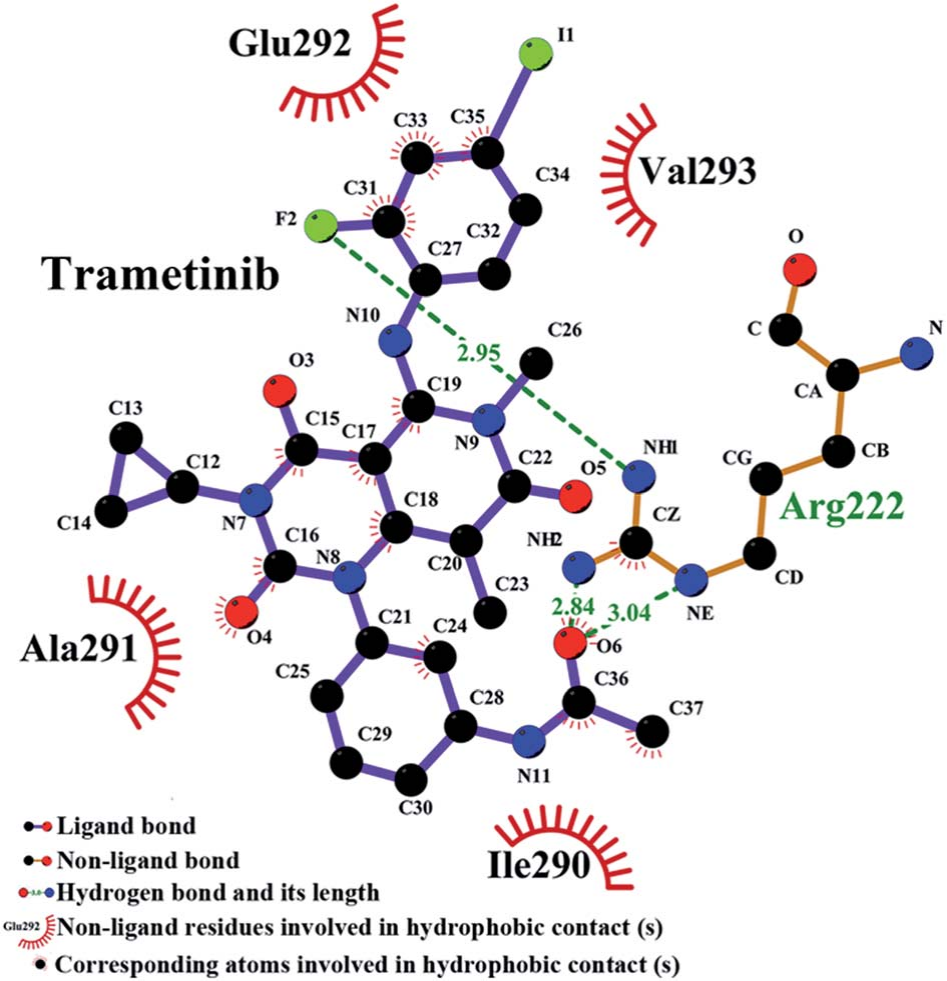
Conformation of the trametinib–HSA complex after 30 ns MD simulation.

The RMSD plots of the free HSA and trametinib–HSA complex are presented in Fig. 7A. The RMSD values of both systems reached equilibrium after 15 ns of MD simulations. The results suggested that both systems reached equilibration after 15 ns of simulations.^41^ Several sampled snapshots of the trametinib–HSA complex in the MD simulation processes are illustrated in Fig. S2.† We can observe that trametinib steadily binds to Sudlow site I. The Rg values are a sign of protein compactness. The Rg values of free HSA and the trametinib–HSA complex (Fig. 7B) were stabilized which suggested that the structure of the HSA in both systems stably folded in 30 ns MD simulation. The mean Rg value was 28.20 Å for free HSA and 28.16 Å for the trametinib–HSA complex. This result suggested that the compactness and folding state of HSA were almost unchanged by trametinib insertion.^42^ The secondary structure percentages of both systems (Fig. 7C and D) were steady, and the values were close. This result suggested that the secondary structure of HSA in both systems was stabilized in the MD simulation processes, and the secondary structure of HSA was unaffected by trametinib insertion.^43^ The final conformation of the trametinib–HSA complex after 30 ns MD simulations is presented in Fig. 8. The trametinib molecule was connected to Arg 222 by three hydrogen bonds and to four amino acid residues (Ile 290, Ala 291, Glu 292, and Val 293) by hydrophobic interactions. The total hydrogen bond energy was 37.27 kJ mol^−1^ and the total hydrophobic interaction energy was 14.01 kJ mol^−1^. The conformation of the trametinib–HSA complex after 30 ns MD simulations was the more appropriate model in the solution than the docking results.

### 3.6 Effect of amino acids on trametinib–HSA interaction

Drug binding to HSA may be affected by other compounds. Amino acids are an important part of blood plasma.^44^ Previous investigators have pointed out the concentrations of circulating amino acids are different and varied for cancer patients.^45,46^ This may affect the binding of trametinib to HSA and further influence the transport and distribution of trametinib in the body.

**Fig. 9:**
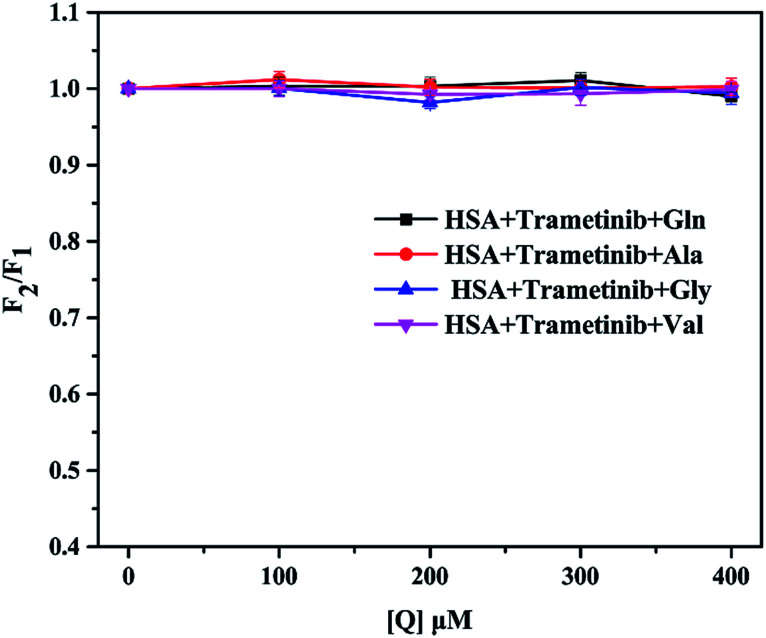
Effect of Gln, Ala, Gly, and Val on the fluorescence intensity of HSA–trametinib.

The concentrations of glutamine (Gln), alanine (Ala), glycine (Gly), and valine (Val) are much higher than those of other amino acids in blood plasma.^47^ Thus, these four amino acids were used as representatives in this study. The four amino acids showed no fluorescence and did not change the fluorescence of HSA in the test condition (Fig. S3†). The molar ratio of trametinib and HSA was maintained at 1 : 1. We selected the fluorescence intensity as a parameter to characterize the binding of trametinib to HSA. The percentage (*I*) of the initial fluorescence was calculated using eqn (9), where *F*_1_ is the fluorescence intensity of the trametinib–HSA complex, and *F*_2_ is the fluorescence intensity of the system after amino acid insertion. The fluorescence intensity of the trametinib–HSA complex was almost unaffected by the various concentrations of the four amino acids (Fig. 9). This result suggests that the trametinib–HSA interaction was almost uninfluenced by the variation of amino acid concentrations.

## 4. Conclusions

In this study, we found that trametinib can bind to HSA. The fluorescence quenching mechanism of trametinib with HSA is static quenching. Trametinib can spontaneously bind to HSA mainly through hydrogen bonding and van der Waals forces. The main binding site for trametinib is the Sudlow site I located in subdomain IIA of HSA. The microenvironment of the chromophore molecules and the structure of HSA were almost unchanged by trametinib binding. The binding process was almost uninfluenced by varying amino acid (Gln, Ala, Gly, and Val) concentrations. The molecular docking and MD simulation results are consistent with the results of the experiment. This study can provide useful information for the pharmacokinetic properties of trametinib.

## Conflicts of interest

There are no conflicts to declare.

## Supplementary Material

RA-008-C7RA12890H-s001
