# Supplementary material for: Combined spectroscopy methods and molecular simulations for the binding properties of trametinib to human serum albumin
Source: RSC Adv. 2018 Jan 26;8(9):4742–9. doi: 10.1039/c7ra12890h (PMC9077761; doi:10.1039/c7ra12890h)
Supplement: RA-008-C7RA12890H-s001 [file RA-008-C7RA12890H-s001.pdf]

Electronic Supplementary Material (ESI) for RSC Advances.
This journal is © The Royal Society of Chemistry 2018

Electronic Supplementary Information (ESI)

Combined spectroscopy methods and molecular simulations on the binding properties of trametinib to human serum albumin

Zili Suo, Qiaomei Sun, Hongqin Yang, Peixiao Tang*, Ruixue Gan, Xinnuo Xiong, and Hui Li*

College of Chemical Engineering Sichuan University, Chengdu 610065, PR China

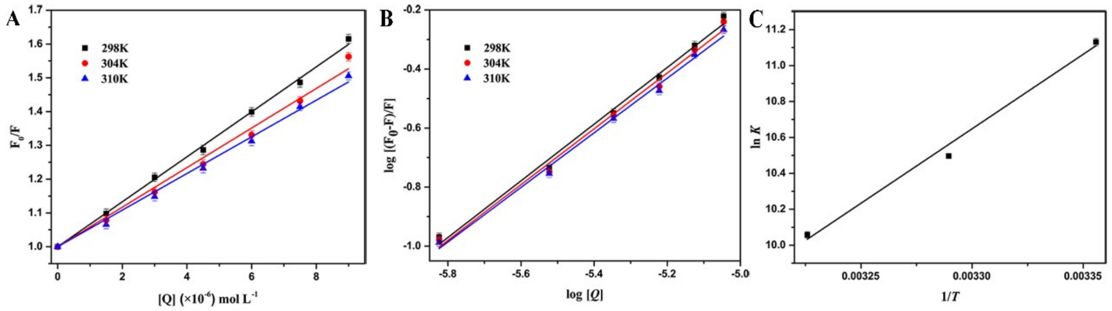

Fig. S1 Stern-Volmer plots (A), the Double-log plots (B) and the van't Hoff plot (C) for the trametinib-HSA interaction.

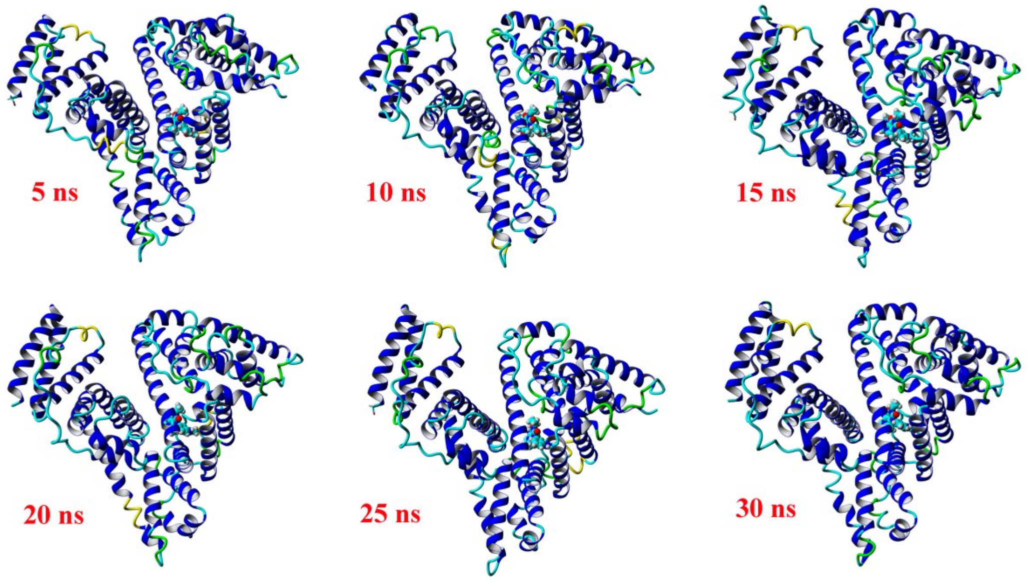

Fig. S2 Several sampled snapshots of trametinib–HSA complex in the MD simulation.

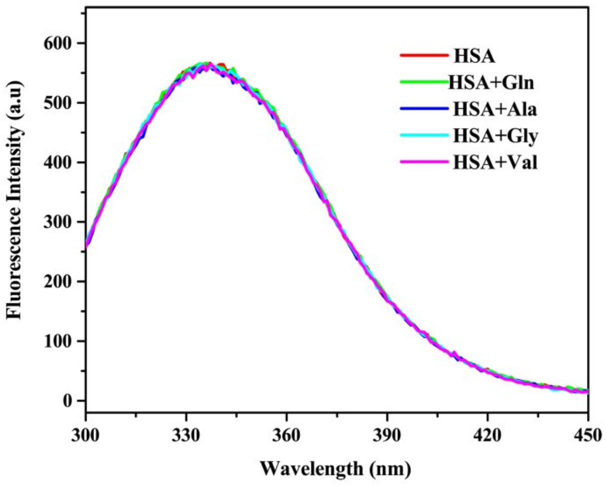

Fig. S3 Fluorescence emission spectra of HSA (2.0 μM) with Gln, Ala, Gly, and Val (400.0 μM).

Table S1 Content of α-helical of HSA under different concentrations of trametinib.

System	$C_{\text{Trametinib}}$ (μM)	α-helical (%)
Trametinib–HSA	0.0	53.25
	4.0	52.43
	8.0	52.89
